# Cell-specific plasticity associated with integrative memory of triple sensory signals in the barrel cortex

**DOI:** 10.18632/oncotarget.25740

**Published:** 2018-07-24

**Authors:** Jing Feng, Wei Lu, Guang-Yan Wang, Zhao-Ming Zhu, Yan Sun, Kaixin Du, Jin-Hui Wang

**Affiliations:** ^1^ Department of Biology, University of Science and Technology China, Hefei, China; ^2^ Institute of Biophysics and University of Chinese Academy of Sciences, Beijing, China; ^3^ University of Chinese Academy of Sciences, Beijing, China; ^4^ Qingdao University, School of Pharmacy, Shandong, China

**Keywords:** learning memory, glutamate, GABA, synapse, barrel cortex

## Abstract

Neuronal plasticity occurs in associative memory. Associative memory cells are recruited for the integration and storage of associated signals. The coordinated refinements and interactions of associative memory cells including glutamatergic and GABAergic neurons remain elusive, which we have examined in a mouse model of associative learning. Paired olfaction, tail and whisker stimulations lead to odorant-induced and tail-induced whisker motions alongside whisker-induced whisker motion. In mice that show this cross-modal associative memory, barrel cortical glutamatergic and GABAergic neurons are recruited to encode the newly learned odor and tail signals alongside the innate whisker signal. These glutamatergic neurons are functionally upregulated, and GABAergic neurons are refined in a homeostatic manner. The mutual innervations between these glutamatergic and GABAergic neurons are upregulated. Therefore, the co-activations of sensory cortices by pairing the input signals recruit their glutamatergic and GABAergic neurons to be associative memory cells, which undergo coordinated refinement among glutamatergic and GABAergic neurons as well as homeostatic plasticity among subcellular compartments in order to drive these cells toward the optimal state for the integrative storage of associated signals.

## INTRODUCTION

Associative memory is presumably critical for cognition, such as associative thinking and logical reasoning [1, 2]. In addition to the associated signals from a sensory modality for intramodal associative memory [3], the brain integrates multiple featured signals from each object that are detected by various sensory modalities [1]. This integration can facilitate the joint storage and the reciprocal retrieval of the associated signals. In associative learning, how cerebral cortices integrates and memorizes these cross-modal signals and distinguishably retrieves them remains to be addressed. In the study of cellular mechanisms underlying associative memory, activity-dependent plasticity at the synapses and the neurons is presumably involved [4–12], and associative memory cells are recruited in the sensory cortices for the integration and storage of the associated signals [13–15]. How associative memory cells in the cerebral cortex for information storage undergo coordinated and homeostatic plasticity remains unclear [16, 17].

Physiological interactions and balances between cortical excitatory and inhibitory neurons are essential for programming brain codes to manage well-organized cognitions [18–20]. How glutamatergic and GABAergic neurons are coordinately refined for the storages of the associated signals remains to be addressed [21, 22]. In addition to associative memory cells to encode two associated signals [14, 23, 24], the cortical neurons can be recruited as associative memory cells that encode triple sensory signals [15]. The storage of multiple signals in individual neurons presumably expands memory volume, strengthens cognition ability and facilitates creative inspiration. Here, we propose to investigate plastic changes in these associative memory cells including glutamatergic and GABAergic neurons.

To the issues above, we have studied the coordinated refinements between barrel cortical glutamatergic and GABAergic neurons with our mouse model of encoding triple sensory signals. To read out cell-specific mechanisms, glutamatergic neurons were genetically labeled by yellow fluorescent protein, and GABAergic neurons were labeled by green fluorescent protein [25]. The confocal cell imaging and electrophysiology were used to analyze the refinements at these synapses and neurons.

## RESULTS

### Barrel cortical neurons are recruited to be associative memory cells to encode triple signals

**Figure 1:**
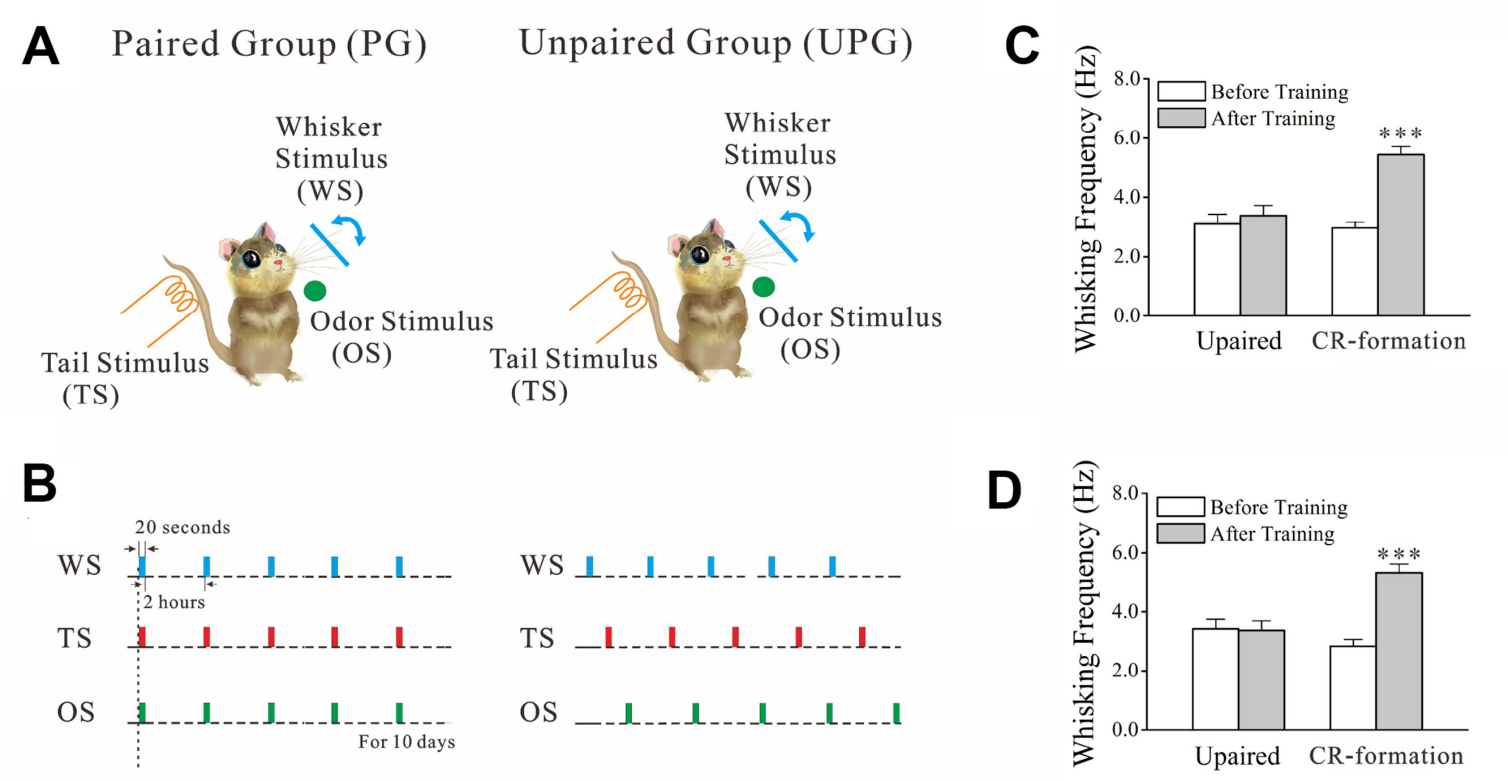
The associations of whisker stimulus (WS), olfactory stimulus (OS) and tail stimulus (TS) lead to odorant-induced and tail-induced whisker motions WS was mechanical vibration at 5 Hz in the intensity to induce whisker fluctuation. TS was a heat touch to the tail at 45 ± 2° C. OS to the noses was butyl acetate to sufficiently evoke olfactory bulb responses. The durations of these stimuli were 20 seconds. (**A**) Training protocols were given to WS/OS/TS-paired group (left panel) and unpaired group (right). (**B**) The paired paradigm was simultaneously giving WS, OS and TS last for 20 seconds, 5 times per day with 2 hour intervals for ten days. The unpaired paradigm was these parameters with at least 5 minutes in intervals among WS, OS and TS. (**C**) Whisking frequencies in response to the OS are 3.42 ± 0.33 Hz before training (white) and 3.37 ± 0.32 Hz and after trainings (gray) from unpaired controls (*n* = 7), and are 2.83 ± 0.34 Hz before training (white) and 5.53 ± 0.30 Hz and after training (gray) from CR-formation mice (*n* = 9, *p* < 0.001, paired *t*-test). (**D**) Whisking frequencies in response to TS are 3.11 ± 0.32 Hz before training (white) and 3.38 ± 0.34 Hz after trainings (gray) from unpaired controls (*n* = 7), and are 2.97 ± 0.20 Hz before training (white) and 5.44 ± 0.28 Hz after training (gray) from CR-formation mice (*n* = 9, *p* < 0.001, paired *t*-test).

Two groups of mice were trained by pairing whisker stimulus (WS), odor stimulus (OS) and tail stimulus (TS) simultaneously as paired group (PG) or by giving these stimulations without pairing as unpaired group (UPG). The protocols used in PG and UPG mice included each training in twenty seconds, five times in two-hour intervals per day for ten days (Figure 1A). After these trainings, the OS and TS induce whisker motions in PG mice, but not UPG mice (Figure 1B), along with whisker-induced whisker motion. Whisking frequency in response to OS (Figure 1C) and TS (Figure 1D) are different significantly before and after training in PG mice, but not in UPG mice. Thus, the association of odor and tail signals with whisker signal causes odorant-induced and tail-induced whisker motions alongside whisker-induced whisker motion, a novel type of cross-modal reflexes (CR). PG mice with CR are named as CR-formation mice.

Whether barrel cortical neurons encoded these multisensory signals was examined by electrophysiological recording. As illustrated in Supplementary Figure 1 of supplementary data, barrel cortical neurons respond to the WS, OS and TS in CR-formation mouse (top panel), but barrel cortical neurons in UPG mouse respond to WS only (bottom panel). Moreover, some glutamatergic and GABAergic neurons in the barrel cortex become to encode such triple associated signals in CR-formation mice, but not in UPG mice (Supplementary Figure 2 in supplementary data). Therefore, glutamatergic and GABAergic neurons in the barrel cortex are recruited to encode the OS and TS signals alongside the innate whisker signal after associative memory forms. In other words, some barrel cortical glutamatergic and GABAergic neurons is recruited as associative memory cells that integrate and store triple signals [15].

In addition to receiving new synapse innervations from the co-activated cortices for barrel cortical neurons to be associative memory cells [1, 15], these barrel cortical glutamatergic and GABAergic neurons may undergo coordinated plasticity, in which the upregulation of glutamatergic neurons and the downregulation of GABAergic neurons facilitate their activities for associative memory cell recruitment to fulfill the integrative memory of multiple signals. This hypothesis has been examined and now is presented below.

### Barrel cortical glutamatergic neurons are upregulated after associative learning

The recruitments of glutamatergic neurons to integrate and encode multisensory signals may be caused by the upregulations of excitatory synaptic inputs as well as the downregulation of inhibitory synaptic inputs, which we examined at YFP-labeled barrel cortical glutamatergic neurons from CR-formation mice vs controls. Morphological change in the spines was analyzed on apical dendrites. Spontaneous excitatory postsynaptic currents (sEPSC) were recorded to assess the change of excitatory synaptic transmission. Spontaneous inhibitory postsynaptic currents (sIPSC) were recorded to assess inhibitory synaptic function.

**Figure 2:**
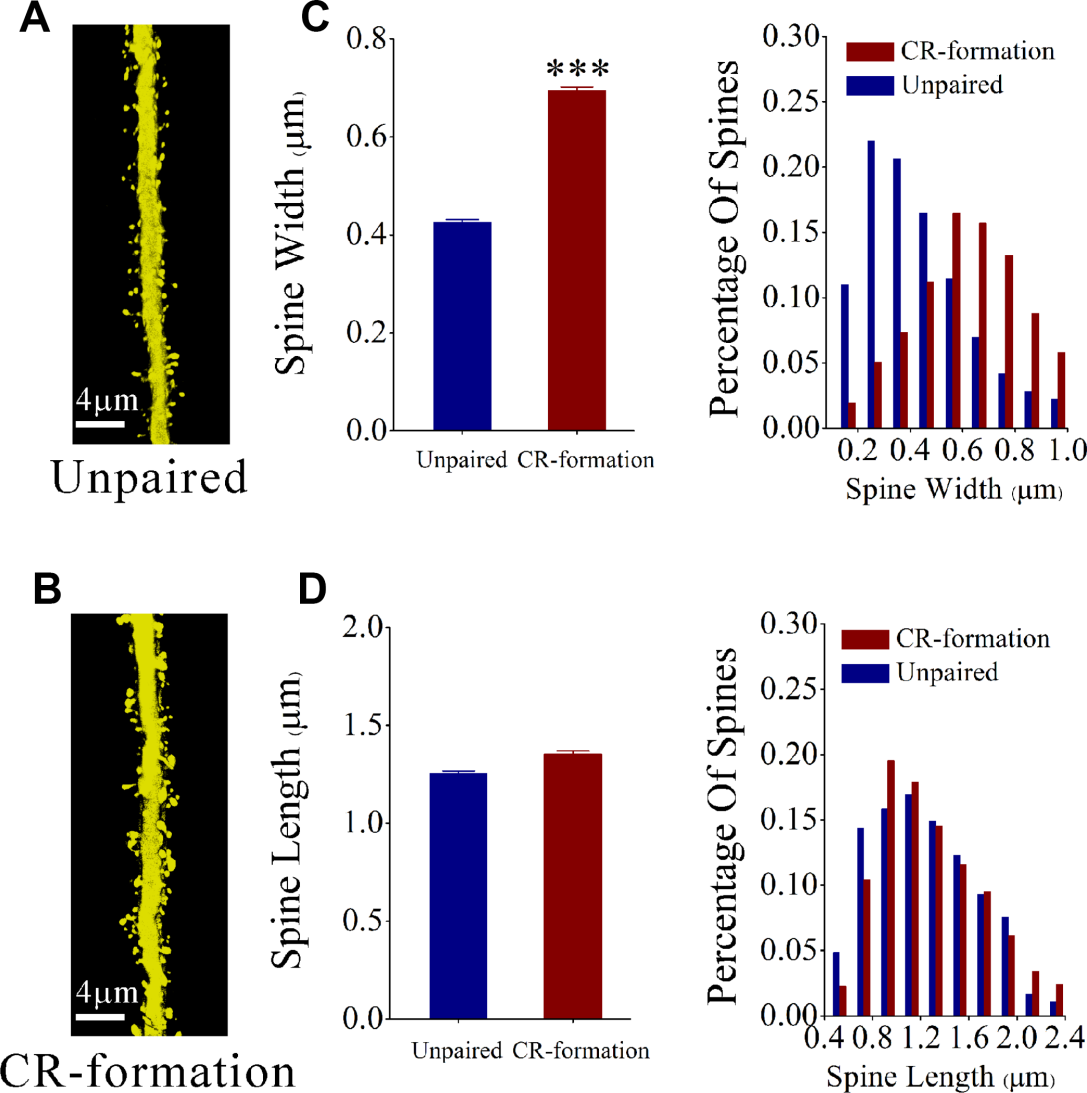
The head of the spines on the apical dendritic of barrel cortical glutamatergic neurons are upregulated after pairing WS, OS and TS (**A**, **B**) The spine volume appears larger on CR-formation neurons (down panel) than unpaired controls (up). (**C**) shows the comparisons of widths of spine head from CR-formations (red bar, *n* = 1643 dendrites on 13 cells from 6 mice) and unpaired controls (blue bar, *n* = 2121 dendrites on 13 cells from 6 mice). The spine head tends to be large in CR-formation mice. (three asterisks, *p* < 0.001, one-way ANOVA). The distribution curve of spine head intuitively show this statistical results in the right panel. (**D**) shows the comparisons of spine lengths from CR-formations (red bar, *n* = 1643 dendrites on 13 cells from 6 mice) and unpaired controls (blue bar, *n* = 2121 dendrites on 22 cells from 6 mice). The spine lengths have no significant change in two group mice (*p* = 0.27, one-way ANOVA). The distribution curve of spine length intuitively show this statistical results in the right panel.

In terms of spine morphology, we have measured head width and length since large head and short neck are presumably functional spines that form the synapses with axonal boutons [12]. After the associative learning, the spine head appears larger and the spine length appears no obvious change in glutamatergic neurons in CR-formation mice (Figure 2A) than those in controls (Figure 2B). Spine widths are 0.69 ± 0.01 μm in CR-formation neurons (red bar in Figure 2C) and 0.42 ± 0.01 μm in control (blue). Spine lengths are 1.35 ± 0.02 μm in CR-formation (red bar in Figure 2D) and 1.25 ± 0.01 μm in control (blue). Spine heads tend to be larger on apical dendrites of CR-formation neurons (*p* < 0.001, *n* = 1643 dendrites of 13 neurons from 6 CR-formation mice and *n* = 2121 dendrites of 22 neurons from control mice, one-way ANOVA). Statistical analysis is intuitively illustrated by the distribution curve of spine head and length (right panel in Figure 2C, 2D). Associative learning makes dendritic spines on glutamatergic neurons being enlarged for receiving new synapse innervation and forming new synapses, which is consistent with suggestions that the enlarged spines play a role in the memory and cognition [12, 26].

**Figure 3:**
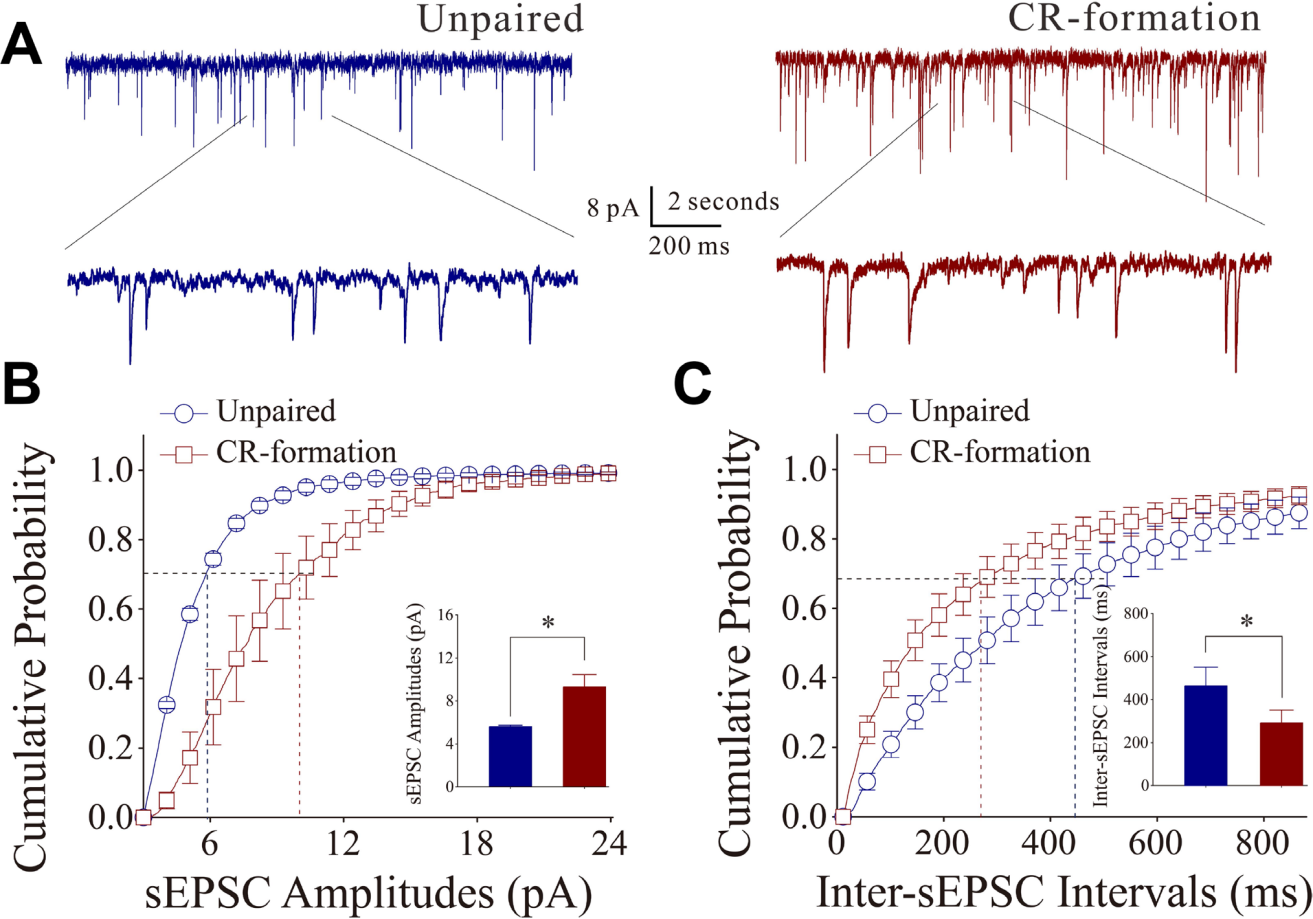
Excitatory synaptic transmission on barrel cortical pyramidal neurons increases after multisensory associative leaning Spontaneous excitatory postsynaptic currents (sEPSC) were recorded on the pyramidal neurons in cortical slices under voltage-clamp (holding potential at –70 mV) in perfusion of 10 μM bicuculline. (**A**) Representative traces of sEPSCs in unpaired control (dark-blue trace in left panel) and CR-formation (dark-red in right). Bottom traces are the expanded waveforms selected from top traces. Calibration bars are 8 pA, 2 second (top) and 200 ms (bottom). (**B**) Cumulative probability curves for sEPSC amplitudes from unpaired control (dark-blue symbols, *n* = 7 from 5 mice) and CR-formation neurons (dark-reds, *n* = 10 from 6 mice). sEPSC amplitudes on barrel cortical glutamatergic neurons at cumulative probability to 67% (CP67) are 5.61 ± 0.13 pA in unpaired controls and 9.27 ± 1.19 pA in CR-formation mice (*p* = 0.02, one-way ANOVA). (**C**) Cumulative probability curves for sEPSC frequency, measured based on inter-event intervals from control (dark-blue symbols, *n* = 7 from 5 mice) and CR-formation (dark-reds, *n* = 10 from 6 mice). Inter-sEPSC intervals on barrel cortical glutamatergic neurons at cumulative probability to 67% (CP67) are 462.8 ± 89.20 ms in unpaired controls and 292.0 ± 59.46 ms in CR-formation mice (*p* = 0.03, one-way ANOVA).

Figure 3A illustrates the functional changes of glutamatergic synapses in cortical slices from CR-formation and control mice. sEPSCs were recorded by whole-cell voltage-clamp at barrel cortical glutamatergic neurons in the presence of 10 µM bicuculline. Figure 3B shows cumulative probability versus sEPSC amplitudes in CR-formation neurons (*n* = 10 from 5 mice) and controls (*n* = 7 from 6 mice). Figure 3C shows cumulative probability versus inter-sEPSC intervals from CR-formation neurons (*n* = 10 from 5 mice) and control cells (*n* = 7 from 6 mice). Cumulative probability curve for sEPSC amplitudes in CR-formation neurons (dark-red symbols, Figure 3B) shifts toward right significantly, compared to that in unpaired controls (dark-blue). sEPSC amplitudes on barrel cortical glutamatergic neurons at 67% cumulative probability are 5.61 ± 0.13 pA in unpaired controls and 9.27 ± 1.19 pA in CR-formation mice (*p* = 0.02, one-way ANOVA). Cumulative probability curve for inter-EPSC interval in CR-formation neurons (dark-red symbols in Figure 3C) shifts toward left significantly, compared to that in unpaired controls (dark-blue). Inter-sEPSC intervals on barrel cortical glutamatergic neurons at 67% cumulative probability are 462.8 ± 89.20 ms in unpaired controls and 292.0 ± 59.46 ms in CR-formation mice (*p* = 0.03, one-way ANOVA). In other words, sEPSC frequency is increased in CR-formation mice. Therefore, barrel cortical glutamatergic neurons in CR-formation mice possess upregulated excitatory synaptic transmission through receiving more new synapse innervations or increased transmitter release as well as receptor responsiveness and number.

**Figure 4:**
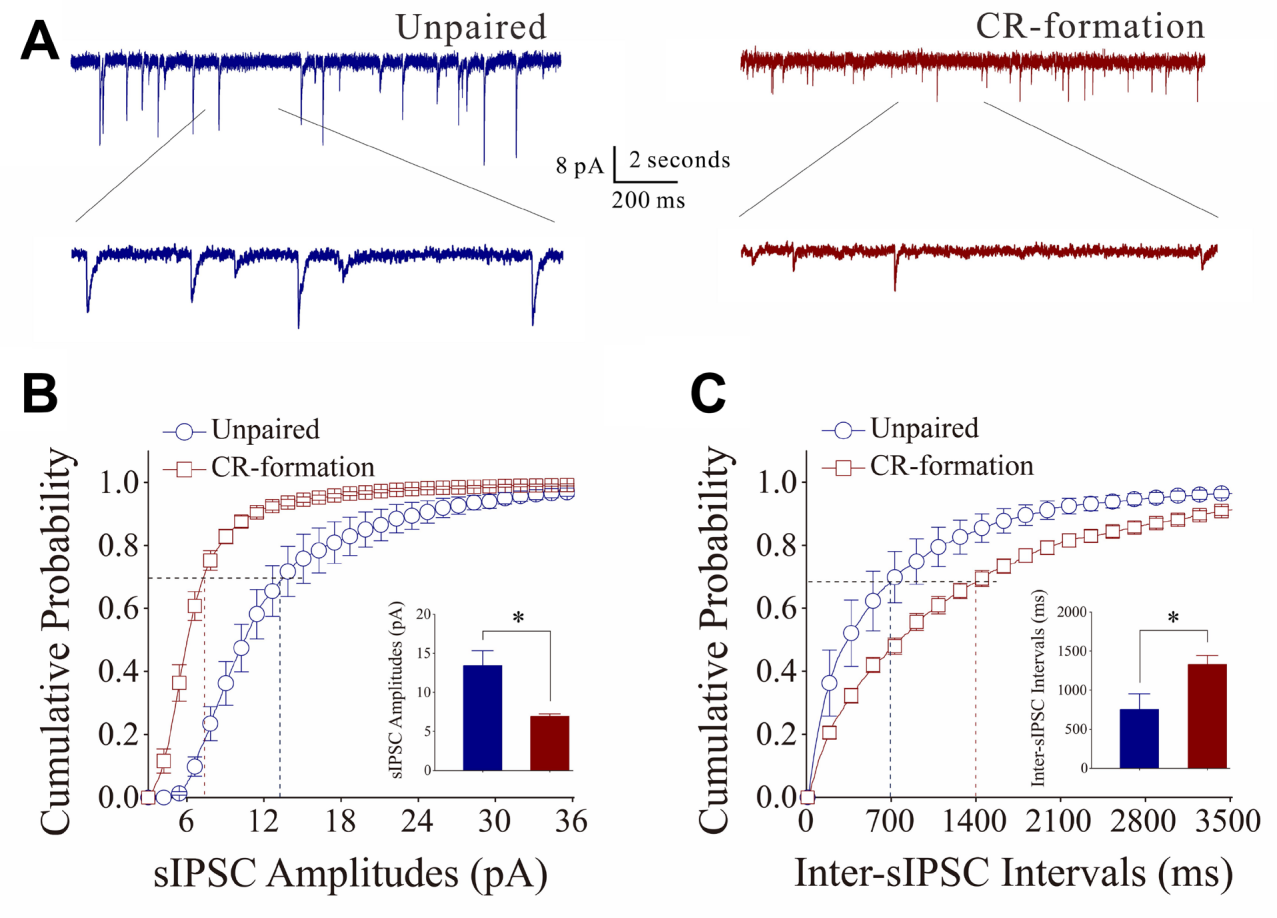
Inhibitory synaptic transmission on barrel cortical pyramidal neurons decreases after multisensory associative leaning Spontaneous inhibitory postsynaptic currents (sIPSC) were recorded on the pyramidal neurons in cortical slices under voltage-clamp (holding potential at –70 mV) in perfusion 10 μM CNQX and 40 μM D-AP5. (**A**) Representative traces of sIPSCs in unpaired control (dark-blue trace in left panel) and CR-formation (dark-red in right). Bottom traces are the expanded waveforms selected from top traces. Calibration bars are 8 pA, 2 second (top) and 200 ms (bottom). (**B**) Cumulative probability curves for sIPSC amplitudes from unpaired control (dark-blue symbols, *n* = 7 from 4 mice) and CR-formation neurons (dark-reds, *n* = 11 from 6 mice). sIPSC amplitudes on barrel cortical glutamatergic neurons at the cumulative probability to 67% (CP67) are 13.45 ± 1.87 pA in unpaired controls and 6.93 ± 0.31 pA in CR-formation mice (*p* = 0.02, one-way ANOVA). (**C**) Cumulative probability curves for sIPSC frequency, measured based on inter-event intervals from control (dark-blue symbols, *n* = 7 from 4 mice) and CR-formation (dark-reds, *n* = 11 from 6 mice). Inter-sIPSC intervals on barrel cortical glutamatergic neurons at the cumulative probability to 67% (CP67) are 746.0 ± 196.15 ms in unpaired controls and 1330.36 ± 113.23 ms in CR-formation mice (*p* = 0.03, one-way ANOVA).

The effect of associative learning on inhibitory synaptic functions on barrel cortical glutamatergic neurons is showed in Figure 4. sIPSCs were recorded by whole-cell voltage-clamp at barrel cortical glutamatergic neurons in the presence of 40 µM APV and 10 µM NBQX. sIPSCs appear lower in CR-formation neurons than in controls (Figure 4A). Figure 4B, 4C shows cumulative probability versus sIPSC amplitudes (4B) and inter-sIPSC intervals (4C) in CR-formation mice (red symbols, *n* = 11 neurons from 4 mice) and controls (blues, *n* = 7 neurons from 6 mice). sIPSC amplitudes on barrel cortical glutamatergic neurons at 67% cumulative probability are 13.45 ± 1.87 pA in unpaired controls and 6.93 ± 0.31 pA in CR-formation mice (*p* = 0.02, one-way ANOVA). Inter-sIPSC intervals on barrel cortical glutamatergic neurons at 67% cumulative probability are 746.0 ± 196.15 ms in unpaired controls and 1330.36 ± 113.23 ms in CR-formation mice (*p* = 0.03, one-way ANOVA). Statistical analyses indicate that sIPSC amplitudes and frequency (1/inter-sIPSC interval) are lower from CR-formation neurons than unpaired controls. Therefore, barrel cortical glutamatergic neurons in CR-formation mice have the downregulated inhibitory synaptic function.

In summary, the spines and excitatory synaptic transmission are upregulated and the inhibitory synaptic transmission is downregulated on barrel cortical glutamatergic neurons after associative memory to multisensory is formed. These changes may facilitate the recruitment and refinement of barrel cortical glutamatergic neurons to be associative memory cells. Then, we investigate plasticity on barrel cortical GABAergic neurons after multisensory associative learning.

### Plasticity at the synaptic inputs of barrel cortical GABAergic neurons in homeostatic manner

To plasticity in synaptic inputs of barrel cortical GABAergic neurons during associative memory, we have analyzed the processes as well as excitatory and inhibitory synaptic transmission on GFP-labeled GABAergic cells in CR-formation and control mice. Neuronal process branches were counted to evaluate the change of their receptive fields. Excitatory synaptic transmission was assessed by recording sEPSCs, and inhibitory synaptic transmission was evaluated by recording sIPSC.

**Figure 5:**
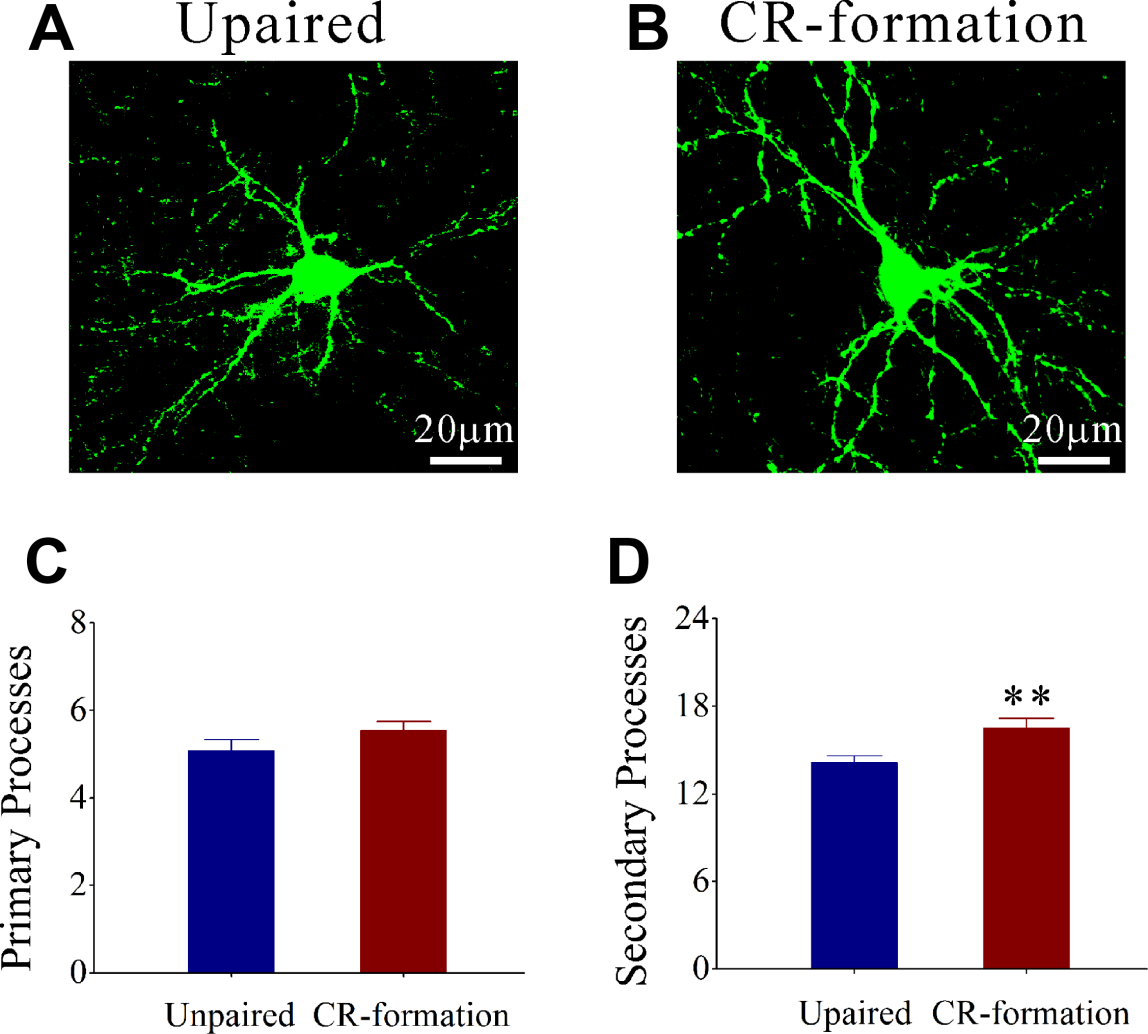
The secondary processes of GABAergic neurons in the barrel cortices increase after multisensory associative learning (**A**, **B**) illustrates that process branches appear denser in CR-formations (B) than controls (A). (**C**) Primary processes per GABAergic neuron seem no significant change in CR-formation mice (dark-red, *n* = 38 cells from 6 mice) and in unpaired controls (dark-blue, *n* = 24 cells from 6 mice; *p* = 0.28, one-way ANOVA). (**D**) The secondary process branches per neuron are higher in CR-formation (dark-red) than in unpaired control mice (dark-blue, two asterisks, *p* < 0.01, one-way ANOVA).

Process branches of GFP-labeled GABAergic neurons were counted in CR-formation and unpaired control mice. Process branches appear denser in CR-formation neurons (Figure 5A) than in controls (Figure 5B). Primary processes per neuron are not statistically changed in CR-formation (5.54 ± 0.20, *n* = 38 neurons from 6 mice) versus unpaired controls (5.08 ± 0.18, *n* = 24 neurons from 6 mice; *p* = 0.28, one-way ANOVA; Figure 5C). Secondary processes per neuron are higher in CR-formation (16.53 ± 0.63, *n* = 38 cells from 6 mice) than controls (14.17 ± 0.32, *n* = 24 cells from 6 mice; *p* < 0.01, one-way ANOVA; Figure 5D). GABAergic neurons have high capacity to receive excitatory inputs after associative memory, which may be used to receive new synapse innervation.

**Figure 6:**
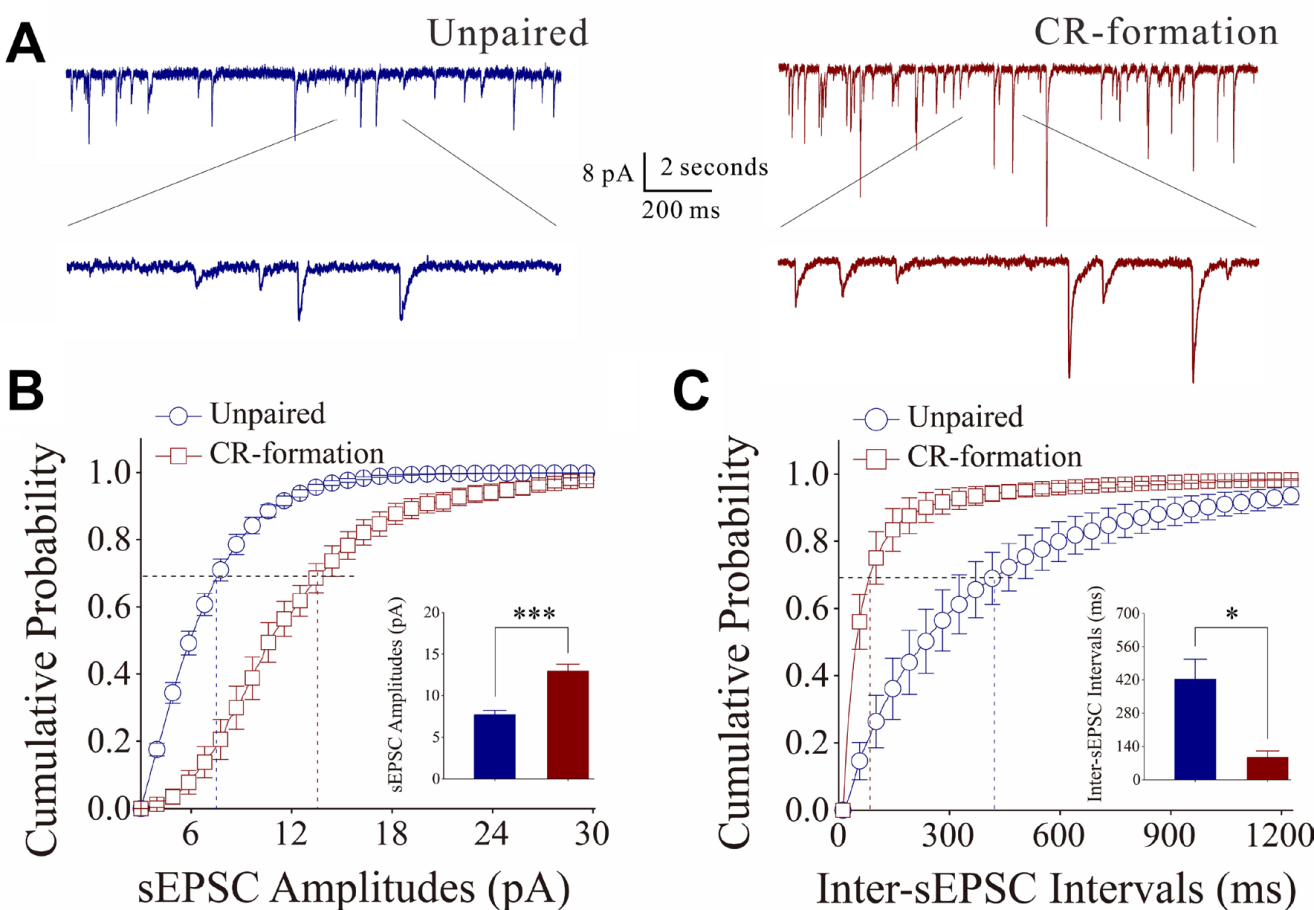
Excitatory synaptic transmission on barrel cortical GABAergic neurons increases after multisensory associative leaning Spontaneous excitatory postsynaptic currents (sEPSC) were recorded on the GABAergic neurons in cortical slices under voltage-clamp (holding potential at −70 mV) in perfusion 10 μM bicuculline. (**A**) Representative traces of sEPSCs in in control (dark-blue trace in left panel) and CR-formation (dark-red in right). Bottom traces are the expanded waveforms selected from top traces. Calibration bars are 8 pA, 2 second (top) and 200 ms (bottom). (**B**) Cumulative probability curves for sEPSC amplitudes from control (dark-blue symbols, *n* = 7 from 6 mice) and CR-formation neurons (dark-reds, *n* = 10 from 6 mice). sEPSC amplitudes on barrel cortical GABAergic neurons at the cumulative probability to 67% (CP67) are 7.69 ± 0.51 pA in unpaired controls and 12.92 ± 0.88 pA in CR-formation mice (*p* < 0.001, one-way ANOVA). (**C**) Cumulative probability curves for sEPSC frequency, measured based on inter-event intervals from control (dark-blue symbols, *n* = 7 from 6 mice) and CR-formation (dark-reds, *n* = 10 from 6 mice). Inter-sEPSC intervals on barrel cortical GABAergic neurons at the cumulative probability to 67% (CP67) are 422.27 ± 85.76 ms in unpaired controls and 93.31 ± 27.54 ms in CR-formation mice (*p* = 0.02, one-way ANOVA).

Figure 6A shows the functional change of excitatory synaptic transmission on barrel cortical GABAergic neurons in cortical slices from CR-formation and control mice. sEPSCs were recorded by using whole-cell voltage-clamp at barrel cortical glutamatergic neurons in the presence of 10 µM bicuculline. Figure 6B illustrates cumulative probability versus sEPSC amplitudes from CR-formation neurons (*n* = 10 from 6 mice) and controls (*n* = 7 from 6 mice). Figure 6C illustrates cumulative probability versus inter-sEPSC intervals from CR-formation neurons (*n* = 10 from 6 mice) and controls (*n* = 7 from 6 mice). Cumulative probability curve for sEPSC amplitude in CR-formation neurons (dark-red symbols) shifts toward right significantly, compared with that in controls (dark-blue). sEPSC amplitudes on barrel cortical GABAergic neurons at 67% cumulative probability are 7.69 ± 0.51 pA in unpaired controls and 12.92 ± 0.88 pA in CR-formation mice (*p* < 0.001, one-way ANOVA). Likewise, cumulative probability curve for inter-sEPSC intervals in CR-formation neurons (dark-red symbols) shifts toward left significantly, i.e., sEPSC frequency in barrel cortical GABAergic neurons increases in CR-formation mice. Inter-sEPSC intervals on barrel cortical GABAergic neurons at 67% cumulative probability are 422.27 ± 85.76 ms in unpaired controls and 93.31 ± 27.54 ms in CR-formation mice (two-sample *t*-test, *P* = 0.02). Therefore, barrel cortical GABAergic neurons receive the increased driving force from excitatory neurons in associative memory.

**Figure 7:**
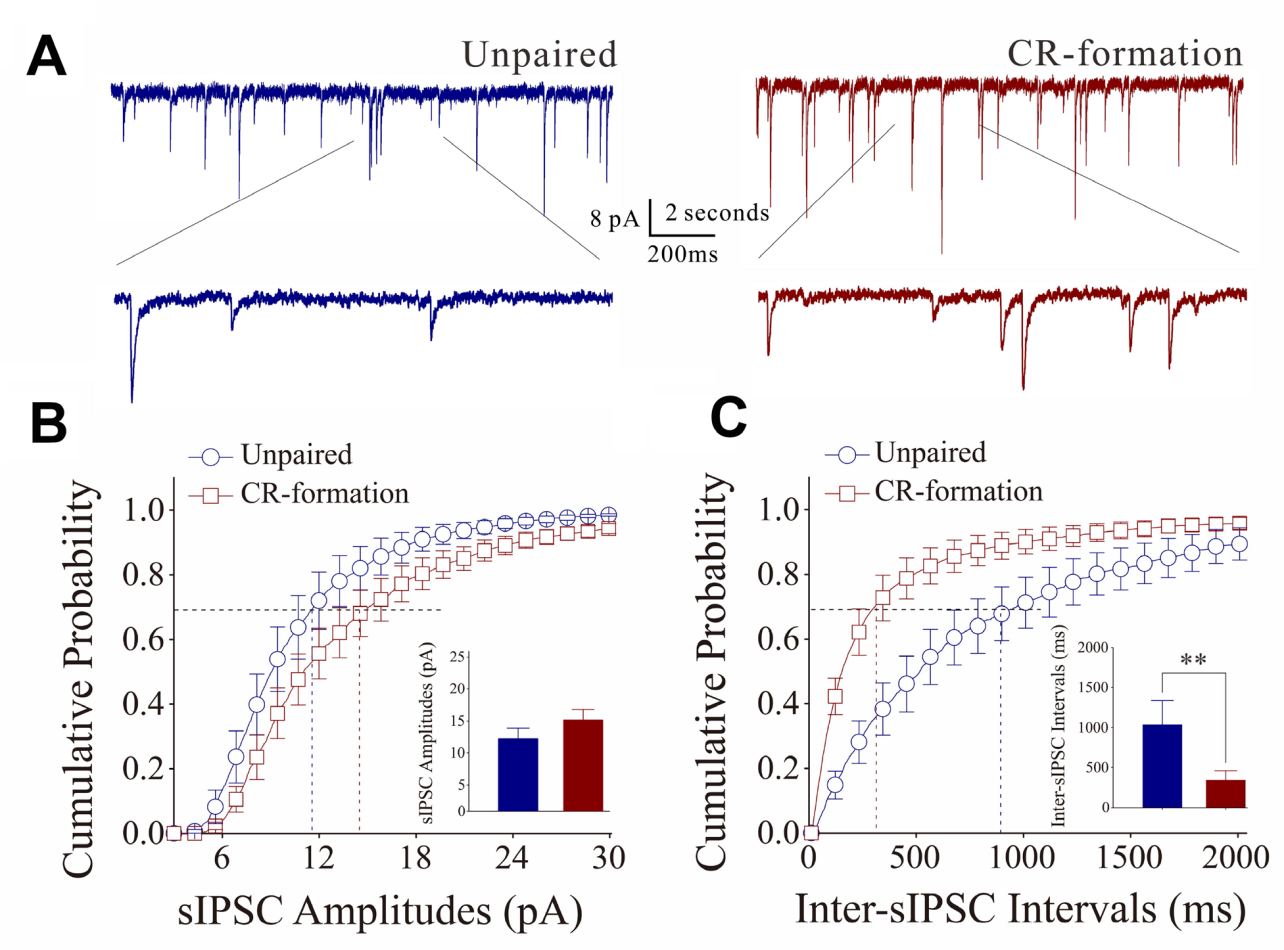
Inhibitory synaptic transmission on barrel cortical GABAergic neurons increases after multisensory associative leaning Spontaneous inhibitory postsynaptic currents (sIPSC) were recorded on the GABAergic neurons in cortical slices under voltage-clamp (holding potential at –70 mV) in perfusion 10 μM CNQX and 40 μM D-AP5. (**A**) Representative traces of sIPSCs in unpaired control (dark-blue trace in left panel) and CR-formation (dark-red in right). Bottom traces are the expanded waveforms selected from top traces. Calibration bars are 8 pA, 2 second (top) and 200 ms (bottom). (**B**) Cumulative probability curves for sIPSC amplitudes from unpaired control (dark-blue symbols, *n* = 7 from 6 mice) and CR-formation neurons (dark-reds, *n* = 10 from 6 mice). sIPSC amplitudes on barrel cortical GABAergic neurons at the cumulative probability to 67% (CP67) are 11.34 ± 1.68 pA in unpaired controls and 14.30 ± 1.64 pA in CR-formation mice (*p* = 0.24, one-way ANOVA). (**C**) Cumulative probability curves for sIPSC frequency, measured based on inter-event intervals from control (dark-blue symbols, *n* = 7 from 6 mice) and CR-formation (dark-reds, *n* = 10 from 6 mice). Inter-sIPSC intervals on barrel cortical GABAergic neurons at the cumulative probability to 67% (CP67) are 1039.4 ± 297.13 ms in unpaired controls and 343.86 ± 116.90 ms in CR-formation mice (*p* < 0.01, one-way ANOVA).

On the other hand, the influence of associative learning on inhibitory synaptic functions on barrel cortical GABAergic neurons is illustrated in Figure 7. sIPSCs were recorded by whole-cell voltage-clamp at GABAergic neurons in the presence of 40 µM APV and 10 µM NBQX. sIPSCs appear higher in CR-formation neurons than in controls (Figure 7A). Figure 7B, 7C shows cumulative probability versus sIPSC amplitudes (Figure 7B) and inter-sIPSC intervals (Figure 7C) in CR-formation mice (red symbols, *n* = 10 neurons from 7 mice) and controls (blue, *n* = 7 neurons from 5 mice). sIPSC amplitudes on barrel cortical GABAergic neurons at 67% cumulative probability are 11.34 ± 1.68 pA in unpaired controls and 14.30 ± 1.64 pA in CR-formation mice (*p* = 0.24, one-way ANOVA). Inter-sIPSC intervals on barrel cortical GABAergic neurons at 67% cumulative probability are 1039.4 ± 297.13 ms in unpaired controls and 343.86 ± 116.90 ms in CR-formation mice (*p* < 0.01, one-way ANOVA). Although there is no significant change in sIPSC amplitudes, sIPSC frequency (1/inter-sIPSC interval) are significantly increased from CR-formation neurons in comparison with unpaired controls. Therefore, barrel cortical GABAergic neurons in CR-formation receive more inhibitory synaptic inputs.

In brief, associative learning upregulates both excitatory and inhibitory synaptic inputs in barrel cortical GABAergic neurons. The upregulated excitatory synaptic inputs and their functions facilitate the driving force to recruit GABAergic neurons as associative memory cells. In the meantime, the upregulated inhibitory synaptic inputs maintain GABAergic neurons not being overly excited.

### Mutual innervation among glutamatergic and GABAergic neurons is upregulated in associative memory

**Figure 8:**
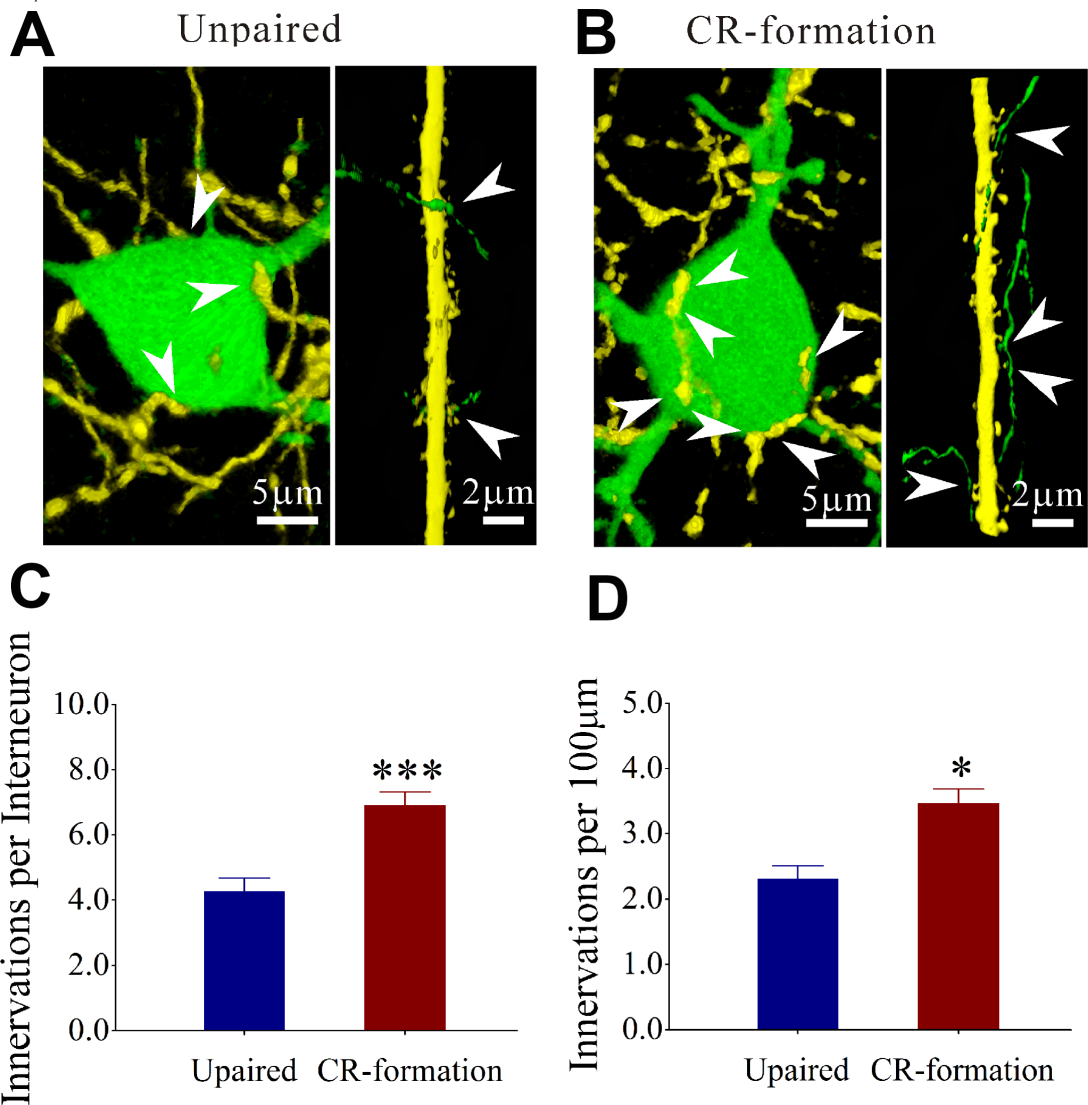
Mutual innervation between excitatory and inhibitory neurons is upregulated after associative learning (**A**) shows YFP-labeled axon terminals on a GFP-labeled GABAergic neuron (left panel) and GFP-labeled axon terminals on YFP-labeled apical dendrite of a glutamatergic neuron (right) from controls. (**B**) shows YFP-labeled axon terminals on a GFP-labeled GABAergic neuron (left panel) GFP-labeled axon terminals on YFP-labeled dendrite of a glutamatergic neuron (right) from CR-formation mice. White arrows indicate their termination. (**C**) shows YFP-labeled axon terminals on each GABAergic neuron in control (dark-blue bar, *n* = 21 from 6 mice) and CR-formation (dark-red, *p* < 0.001, *n* = 37 from 6 mice, one-way ANOVA). (**D**) shows GFP-labeled axon terminals per 100 μm on YFP-labeled apical dendrites of glutamatergic neurons in controls (dark-blue bar, *n* = 15 from 6 mice) and CR-formations (dark-red, *p* < 0.05, *n* = 16 from 6 mice, one-way ANOVA).

In addition to excitatory and inhibitory synapses, the interactions between glutamatergic and GABAergic neurons were investigated by counting YFP-labeled axon terminals on GFP-labeled GABAergic neurons and GFP-labeled axon terminals on YFP-labeled apical dendrites of glutamatergic neurons (Figure 8). Figure 8A illustrates the interactions between glutamatergic and GABAergic neurons in unpaired mice, while Figure 8B illustrates that in paired mice. YFP-labeled axon terminals on the soma of GABAergic neuron are 4.27 ± 0.41 in controls (dark-blue bar in Figure 8C, *n* = 21 from 6 mice) and 6.92 ± 0.41 in CR-formations (dark-red, *n* = 37 from 6 mice; *p* < 0.001, one-way ANOVA). GFP-labeled axon terminals per 100 μm dendrite on the glutamatergic neuron are 2.3 ± 0.26 in controls (dark-blue bar in Figure 8D, *n* = 15 from 6 mice) and 3.45 ± 0.30 in CR-formations (dark-red, *n* = 16 from 6 mice; *p* < 0.05, one-way ANOVA). Mutual innervations between glutamatergic and GABAergic neurons are upregulated during associative memory.

## DISCUSSION

In the mice that express odorant-induced and tail-induced whisker motions (Figure 1), glutamatergic and GABAergic neurons in barrel cortices are recruited as associative memory cells that encode the newly learned odor and tail signals alongside innate whisker signal. In glutamatergic neurons, dendritic spines and excitatory synaptic inputs are upregulated (Figures 2, 3) and inhibitory synaptic inputs are downregulated (Figure 4), which may benefit their recruitments to be associative memory cells to store specific integrative signals as well as drive them to optimal state for sensitively integrating associative signals and for efficiently activating the downstream neurons in memory presentation. In GABAergic neurons, both excitatory and inhibitory synaptic inputs are upregulated in a homeostatic manner (Figures 5, 7), which may promote their recruitment to be associative memory cells and prevent neuronal overexcitation. An enhanced mutual innervation between glutamatergic and GABAergic neurons (Figure 8) maintains homeostasis in local neural networks.

**Figure 9:**
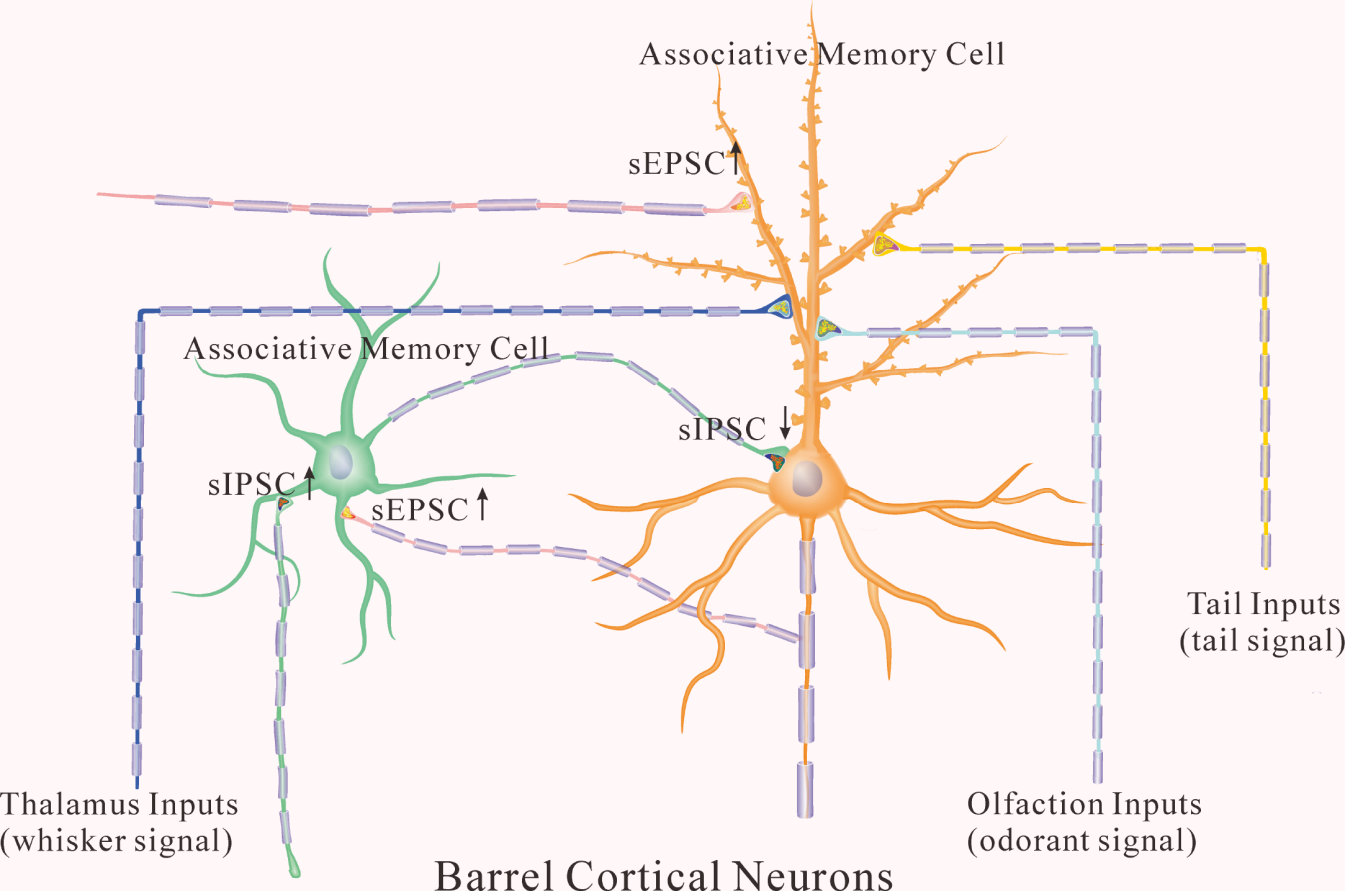
The coordinated recruitment and refinement of barrel cortical glutamatergic and GABAergic neurons set up their function state for information storage In addition to receiving whisker signal from the thalamus, associative memory cells in the barrel cortex receive odor signal from the piriform cortex and tail signal from cortical tail region after multisensory associative learning. In the glutamatergic neurons (orange), their dendritic spines are enriched, their excitatory synaptic transmissions are upregulated, and their receiving of inhibitory synaptic transmission is downregulated for their recruitments to be associative memory cells. In the GABAergic neurons (green), their excitatory synapses and receptive fields are upregulated, which facilitate the recruitment of GABAergic neurons to be associative memory cells. In the meantime, inhibitory synaptic activity on GABAergic neurons is also increased, which keeps these neurons away from overexcitation.

In terms of plasticity at barrel cortical glutamatergic neuron, our study shows the upregulation of excitatory synapses and the downregulation of inhibitory synapses. The coordination among these subcellular compartments makes glutamatergic neurons to be more excitable (Figure 9), which allows the excitatory driving force from new synapse innervations of piriform and S1-tail cortices [15, 27] to recruit them as associative memory cells as well as to refine them with the upregulated ability to encode digital spikes [28–30] for memory formation. In the meantime, the coordination among these synapses boost neuronal sensitivity to input signals and neuronal capability to activate the downstream neurons for behavioral reactions and memory presentations during information retrieval. In addition, the increased inhibitory innervation and the decreased inhibitory synaptic transmission on glutamatergic neurons maintain their functional homeostasis.

To plasticity at barrel cortical GABAergic neurons, our results indicate the upregulations of their excitatory synapses and receptive fields. This increase of excitatory inputs facilitates the recruitment of GABAergic neurons to be associative memory cells. In the meantime, inhibitory synaptic activity on GABAergic neurons is also increased, which maintains them away from overexcitation. On the other hand, their synaptic outputs to inhibit target neurons are decreased, which facilitates the recruitment of other barrel cortical neurons for information storage. This data is consistent to that a disinhibition of neural circuits occurs in fear memory [22]. As GABAergic neurons fire high frequency spikes and are vulnerable to the energy cost [31, 32], their functional downregulation by intensive activity in associative learning may be one of initial steps to trigger the recruitment of associative memory cells under the physiological condition. If their intensive activities plus stressful internal environment may lead to neurological and psychiatric diseases [33–37].

Our result indicates that homeostatic plasticity coordinated among subcellular compartments [38] is involved in associative memory. For instance, the increases of excitatory synaptic inputs and inhibitory synaptic outputs in GABAergic neurons work for maintaining neuronal homeostasis through a coordination of these subcellular compartments. Homeostatic plasticity can also be fulfilled by a coordination of synapse innervation and function. For example, inhibitory synapse function decreases and inhibitory synapse innervation increases in glutamatergic neurons, which prevents the disability of inhibitory synapses to influence these neurons. As we noted, an interesting phenomenon about the output of GABAergic neurons is that inhibitory synapses are downregulated on glutamatergic neurons and upregulated on GABAergic neurons. Because GABAergic neurons are more active than glutamatergic neurons [18–20], axon branches and their synapses onto GABAergic cells are functionally upregulated whereas axon branches and their synapses onto glutamatergic cells are functionally downregulated, which indicates the role of functional compatibility between presynaptic and postsynaptic partners [39, 40] in memory formation.

In terms of recruiting associative memory cells and their coordination, we propose the following molecular and cellular processes. The co-activation of barrel, S1-tail and piriform cortices induces epigenetic alternation. The upregulated miRNAs knock down their target genes, or vice versa. The altered expression of target genes facilitates axonal growth, new synapse innervations and excitatory synapse function, as well as attenuates inhibitory synaptic function. Such cellular changes lead to the coordinated recruitment and refinement of glutamatergic and GABAergic neurons to be associative memory cells. This assumption is supported by our current observations that anti-miRNA-324/-133a attenuate memory retrieval, associative memory cells and synapse innervations through Ttbk1 and Tet3 [15, 27, 41, 42]. The consistent results by applying molecular, functional and morphological approaches strengthen the conclusion reliability of our studies.

Cognitive processes, such as logical reasoning, associative thinking, comparison and computation, require the associated retrievals of pair-stored signals and events from different groups of associative memory cells. These groups of associative memory cells may complete the retrievals of these pair-stored signals based on the pair-by-pair sequence of multiple-grades or on the sharing of common signal in such pair-stored signals. In this regard, the newly wired axon circuit among different brain areas and the newly formed synapses in neural circuits are essential for the communication of associative memory cells in cognitive processes. Our results reveal that the mutual innervations between sensory cortices and the recruitment of associative memory cells in these areas [14, 24, 27] constitute the bases of associative memory and cognitive processes.

Associative memory cells for cross-modal associative memory in sensory cortices (i.e., primary associative memory cells) have following features. In addition to synapse innervations from innate sensory inputs, associative memory cells in the sensory cortex receive new synapse innervations from other co-activated sensory cortices that encode such sensory inputs for their primary integration and storage. They encode multi-associated signals including the innate signal and newly learned signals from external environments. Their axons project and innervate onto the neurons in downstream brain areas relevant to behaviors, cognition and emotion allowing their downstream neurons to encode these associated signals (i.e., secondary associative memory cells) and to initiate memory presentations. The number of the recruited associative memory cells and their upregulated refinements are proportional to memory strength and maintenance. The activation of associative memory cells influences logical reasoning and associative thinking. Their recruitments are controlled by epigenetics-regulated genes and proteins relevant to memory through a chain reaction of intensive spikes and microRNA expression alteration. Cognitive processes, such as associative thinking, logical reasoning, imagination and computation, activate primary and secondary associative memory cells to induce their axon projections and synapse innervations onto neurons in cognitive brain areas for the integration and storage of these endogenous signals, leading to cognition-related memories. The recruitment of associative memory cells and their plasticity influence memory-related physiological and pathological processes [1, 16]. In the storage and retrieval of associated signals, the working principle for associative memory cells is based on their receptions to innate and new synapse inputs, their abilities to convert synaptic analog signals into digital spikes for encoding associated signals and their abilities to output sequential spikes [28–30, 40] that will drive behavior-, cognition- and emotion-related brain regions in memory presentation. Therefore, synapse inputs to associative memory cells determine the specificity of memory contents. The activity power and plasticity at associative memory cells as well as their input and output partners may set up the strengths of information storage and memory presentation. For instance, barrel cortical neurons receive new synapse innervations from the piriform cortex after associative learning, in addition to innate input from the thalamus. Synapse activities induced by odor stimulus drive barrel cortical neurons toward the threshold of firing spikes under the condition of basal thalamic input, and their spikes further activate motor cortical neurons for odorant-induced whisker motion.

Learning to environmental associative signals evokes the recruitment and refinement of primary associative memory cells in sensory cortices. Cognitive processes, such as logical reasoning, associative thinking and so on, will generate endogenous associated signals that are memorized at secondary associative memory cells in the behavior-, emotion- and/or cognition-related brain areas through associated synapse innervations [1]. This hypothesis has been examined in our current study. For instance, after cross-modal associative memory forms, some neurons in the prefrontal cortex are able to encode whisker and olfactory signals, which are recorded by local field potentials and two-photon cellular imaging *in vivo*. These individual neurons in the prefrontal cortex receive synapse innervations from barrel and piriform cortices, where are injected by GFP- and RFP-tagged AAVs, respectively. Our study provides morphological and functional evidence for the recruitment of secondary associative memory cells in the prefrontal cortex, which are used for cognitions. Once multiple signals are transferred to secondary associative memory cells for the storage, they can be recalled spontaneously and during thinking as long as the prefrontal cortex is activated to the threshold.

## MATERIALS AND METHODS

All experiments were performed in accordance with the guidelines by Administration Office of Laboratory Animals at Beijing China. All experiment protocols were approved by Institutional Animal Care Unit Committee in the Administration Office of Laboratory Animals at Beijing China (B10831).

### Mouse model of associative memory

To analyze cell-specific mechanism for associative memory we used C57 Thy1-YFP/GAD67-GFP mice [25] whose glutamatergic neurons were genetically labeled by yellow fluorescent protein (YFP) and GABAergic neurons were labeled by green fluorescent protein (GFP).

### A mouse model of conditioned reflexes

Strain C57 male mice in postnatal day 20 with the intact and symmetric whiskers were divided into two groups that received different treatments (Figure 1A) in whisker stimulus (WS, 5 Hz mechanical stimulus), odor stimulus (OS, butyl acetate) and tail stimulus (TS, the heat plate to touch the tail around 45° C). The trainings included the simultaneous pairing of the OS, TS and WS (paired group, PG) or the un-pairing of the WS, OS and TS (unpaired group, UPG; intervals among the WS, TS and OS about 5 minutes). The WS, TS and OS were given by multiple-sensory modal stimulator (MSMS, ZL201410499466), where their intensity, duration and interval were precisely set. The OS was given by switching on a butyl acetate-contained tube and generating a small liquid drop on its tip in front of the mouse noses without air pressure. The intensity of butyl acetate OS was enough to induce the responses of olfactory bulb neurons detected by two-photon imaging [14]. The WS to the assigned whiskers was given to the contralateral side (right-side) of barrel cortices that were studied in cell imaging and electrophysiology. The WS intensity suitably triggered whisker fluctuations after the end of stimulation, i.e., whisker-induced whisker motion [14]. The TS to the mice was given by using a heat plate that touched to the distal ends of their tails. The TS intensity was about 45 ± 2° C that was sufficient to evoke mouse tail swing away from this heat plate within 10 seconds. This temperature did not make the injury of thick skin on the tail. These parameters to train each mouse in PG and UPG by the WS, TS and OS were twenty seconds each training and five times every day in intervals of two hours for ten days (Figure 1). This training period was based on a fact that the onset of odorant-induced whisker motion reached the plateau level around ten days [14]. Stimulus intensities, duration and frequency were precisely controlled by this MSMS, which were fixed in each trial for the mice. During the training, each of the mice was placed in a home-made cage, in which their running and movement were restricted, but their bodies and arms extended freely. There were no circadian disturbance and stressful conditions, such as the noise, light, unusual odor and motions from experimenters. Mice were placed into the cages for ten minutes every day about one week to have them habituated to experimental conditions before the training, and placed in the cages for five minutes before each training for their quiet adaptation during the training. These cares were also used in the odor-test and the tail-test (please see below; [14]).

Mouse whisker motion tracks were monitored by digital video camera (240 fps). All images were digitized (50 Hz) and converted into whisker motion traces. Whisker motions were quantified by the software self-programed in Matlab, and ImageJ (version. 1.47; the National Institute of Health, USA), including the whisking frequency and fluctuation magnitude. The fluctuation magnitudes were defined as the absolute changes of whisking angles [43]. The responses of mouse whiskers to the odor-test (butyl acetate toward the noses for 20 seconds) and to the heat-test to the tail (45° C) were recorded before the training and one hour after the end of each training day up to day ten to quantify the onset time and levels of odorant-induced whisker motion and tail-induced whisker motion (cross-modal reflex, CR). Odorant-induced and tail-induced whisker motions were accepted if their whisker motions met the criteria below. The patterns of odor-induced and tail-induced whisker motions were similar to the typical whisker motions induced by the WS [14], but not spontaneous low amplitude whisking. Whisking frequencies increased significantly, compared with control and before the training. The OS- or TS-induced whisker motion was originally induced by the WS. In other words, the odor signal or tail signal induced the recall of whisker signal and led to whisker motion, i.e., CR-formation. It is noteworthy that OS-induced whisker motion is not related to mouse sniffing, since the sniffing alters the baseline of whisker motion, which is not a case in our data. Whisking frequency is also greater than the sniffing, and all of the mice do not show the sniffing induced by the OS-test.

The “assigned whiskers” were long whiskers (such as arcs 1∼2) on the same side and same rows that were assigned for the training by mechanical whisker stimuli in the PG and UPG as well as for the odor-test and tail-test in all mice. Their corresponding barrels were studied in field potential recording and two-photon cell imaging. We did not trim short whiskers since a whisker trimming raised the excitability of barrel cortices [25].

### Brain slices and neurons

Cortical slices (400 µm) were prepared from the mice of CR-formation and unpaired controls. They were anesthetized by inhaling isoflurane and decapitated by a guillotine. The slices were cut by Vibratome in the oxygenated (95%O_2_/5%CO_2_) artificial cerebrospinal fluid (ACSF), in which the chemical concentrations (mM) were 124 NaCl, 3 KCl, 1.2 NaH_2_PO_4_, 26 NaHCO_3_, 0.5 CaCl_2_, 4 MgSO_4_, 10 dextrose, and 5 HEPES, pH 7.35 at 4° C. The slices were held in the oxygenated ACSF (124 NaCl, 3 KCl, 1.2 NaH_2_PO_4_, 26 NaHCO_3_, 2.4 CaCl_2_, 1.3 MgSO_4_, 10 dextrose, and 5 HEPES, pH 7.35) at 25° C for 2 hours. The slices were transferred to submersion chamber (Warner RC-26G) that was perfused with the oxygenated ACSF at 31° C for whole-cell recording [44].

Electrophysiological recordings on the neurons in layers II-III of the barrel cortex were conducted under DIC-fluorescent microscope (Nikon FN-E600, Japan). The wavelength at 488 nm excited GFP, and the wavelength at 575 nm excited YFP. GABAergic neurons showed basket shape and fast spiking with less adaptation in spike amplitudes and frequency [45–47]. Glutamatergic neurons showed pyramidal shape and regular spikes with the adaptation of spike amplitudes and frequency [36]. Cerebral slices were coronal sections including the barrels correspondent to the projection from long whiskers that were stimulated in pairing WS and OS training.

### Whole-cell recording

Cortical neurons were recorded by MultiClamp-700B amplifier in voltage-clamp for their synaptic activities. Electrical signals were inputted into pClamp-10 (Axon Instrument Inc, CA USA) for data acquisition and analyses. Output bandwidth in this amplifier was 3 kHz. The pipette solution for studying excitatory synapses included (mM) 150 K-gluconate, 5 NaCl, 5 HEPES, 0.4 EGTA, 4 Mg-ATP, 0.5 Tris-GTP and 5 phosphocreatine (pH 7.35; [48, 49]). The solution for studying inhibitory synapses contained (mM) 130 K-gluconate, 20 KCl, 5 NaCl, 5 HEPES, 0.5 EGTA, 4 Mg-ATP, 0.5 Tris–GTP and 5 phosphocreatine [17, 50]. Pipette solutions were freshly made and filtered (0.1 μm), osmolarity was 295∼305 mOsmol and pipette resistance was 5∼6 MΩ.

The functions of GABAergic neurons were assessed based on their active intrinsic properties and inhibitory outputs [32]. The function status of their inhibitory output was evaluated by recording spontaneous inhibitory postsynaptic currents (sIPSC) under the voltage-clamp on glutamatergic neurons in the presence of 10 µM 6-Cyano-7-nitroquinoxaline-2,3-(1H,4H)-dione (CNQX) and 40 µM D-amino-5-phosphonovanolenic acid (D-AP5) in ACSF to block ionotropic glutamate receptors [10, 51, 52]. 10 µM bicuculline was washed onto the slices at the end of experiments to test whether synaptic responses were mediated by GABA_A_R, which blocked sIPSCs in our experiments. The series and input resistances in all of the neurons were monitored by injecting hyperpolarization pulses (5 mV/50 ms), and calculated by voltage pulses versus instantaneous and steady-state currents. It is noteworthy that pipette solution with the high concentration of chloride ions makes the reversal potential to be –42 mV. sIPSCs will be inward when the membrane holding potential at –65 [50, 52].

The functions of glutamatergic neurons were assessed based on their active intrinsic property and excitatory outputs [32]. The function status of their excitatory output was evaluated by recording spontaneous excitatory postsynaptic currents (sEPSC) on GABAergic or glutamatergic neurons in presence of 10 µM bicuculline in ACSF to block ionotropic GABA receptors [32]. 10 µM CNQX and 40 µM DAP-5 were added into ACSF perfused onto the slices at the end of experiments to examine whether synaptic responses were mediated by GluR, which blocked EPSCs in our experiments. The series and input resistances for all of the cells were monitored by injecting hyperpolarization pulses (5 mV/50 ms), and calculated by voltage pulses versus instantaneous and steady-state currents [16, 53].

The recording of spontaneous synaptic currents, instead of the evoked synaptic currents, is based on the following reasons. sEPSC and sIPSC amplitudes represent the responsiveness and the densities of postsynaptic receptors. The frequencies imply the probability of transmitter release from an axon terminal and the number of presynaptic axons innervated on the recorded neuron. These parameters can be used to analyze presynaptic and postsynaptic mechanisms about the neuronal interaction. The evoked postsynaptic currents cannot separate these mechanisms. We did not add TTX in the ACSF to record miniature postsynaptic currents as we had to record neuronal excitability. As the frequency of synaptic activities was less than those of sequential spikes and the spontaneous spikes were never recorded on the neurons in our cortical slices, sIPSCs and sEPSCs were not generated from spontaneous action potential. The synaptic events in our recording are presumably miniature postsynaptic currents. This point is granted by a single peak of postsynaptic currents in our study [33, 36, 37].

Data were analyzed if the recorded neurons had the resting membrane potentials negatively more than -60 mV, and action potential amplitudes more than 90 mV. The criteria for the acceptance of each experiment also included less than 5% changes in resting membrane potential, spike magnitude, and input resistance throughout each experiment. Input resistance was monitored by measuring cellular responses to hyperpolarization pulse at the same values as the depolarization that evoked action potentials. To estimate the effects of associative learning on the neuronal spikes and synaptic transmission, we measured sEPSC, sIPSC, ISI, ARP and Vts under the conditions of control and associative memory, which were presented as mean ± SE. The comparisons of these data before and after associative learning were done by *t*-test.

### Cellular morphological imaging in the barrel cortices

The control and CR-formation mice were anesthetized by intraperitoneal injections of sodium pentobarbital, and perfused by 4% paraformaldehyde in 0.1 M phosphate buffer solution (PBS) into left ventricle until their bodies were rigid. The brains were quickly isolated and fixed in 4% paraformaldehyde PBS for additional 24 hours. The cerebral brains were sliced in a series of coronal sections at 100 μm, which included the barrels correspondent to the projection from long whiskers that were stimulated in pairing WS and OS training. These slices were rinsed by PBS for three times, air-dried and cover-slipped. In order to clearly show three-dimension images for new synapses in the barrel cortex, we placed the brain slices into a solution (Sca/eA2) for a few hours to make them transparency [54]. The images for YFP-labeled glutamatergic neurons and GFP-GABAergic neurons in cortical layers II∼III were photographed under the confocal microscopy with oil lens (Plan Apo VC 60X, 1.4NA; Nikon A1R plus, Tokyo, Japan). The excite wavelength was 488 nm for GFP and YFP. Although the peaks of GFP and YFP emission wavelengths are closely at 510 and 525 nm, respectively, we scanned the images of such neurons through setting the optical grating in 505∼515 nm for GFP and the optical grating in 545∼555 nm for YFP, to separate their fluorescent images [16]. In the confocal imaging, the resolution was 0.05 μm per pixel, the minimal pixels for the measured spines were at least 9∼10 in the line. Spines were the protrusion extended from dendrites. In the analysis of dendritic spines, their head width and length from primary processes (branches from somata) of pyramidal neurons were measured [55]. In terms of morphological interaction between glutamatergic and GABAergic neurons in the mouse barrel cortex, mutual innervations between these cells were measured by counting the contacts of presynaptic boutons with postsynaptic neurons and dendritic spines [12]. The quantifications were conducted by ImageJ (version 1.47; National Institute of Health, USA). The analyses of processes and spines were given in the method of our previous study [25].

### Statistical analyses

The paired *t*-test was used in the comparisons of the experimental data before and after associative learning, as well as the neuronal responses to whisker stimulus and odorant stimulus in each of the mice. One-way ANOVA was applied to make the statistical comparisons in the changes of neuronal activities and morphology between control and CR-formation groups.

## SUPPLEMENTARY MATERIALS FIGURES


