# Supplementary material for: Cell-specific plasticity associated with integrative memory of triple sensory signals in the barrel cortex
Source: Oncotarget. 2018 Jul 24;9(57):30962–78. doi: 10.18632/oncotarget.25740 (PMC6089555; doi:10.18632/oncotarget.25740)
Supplement: Supplementary file 1 [file oncotarget-09-30962-s001.pdf]

Cell-specific plasticity associated with integrative memory of triple sensory signals in the barrel cortex

SUPPLEMENTARY MATERIALS

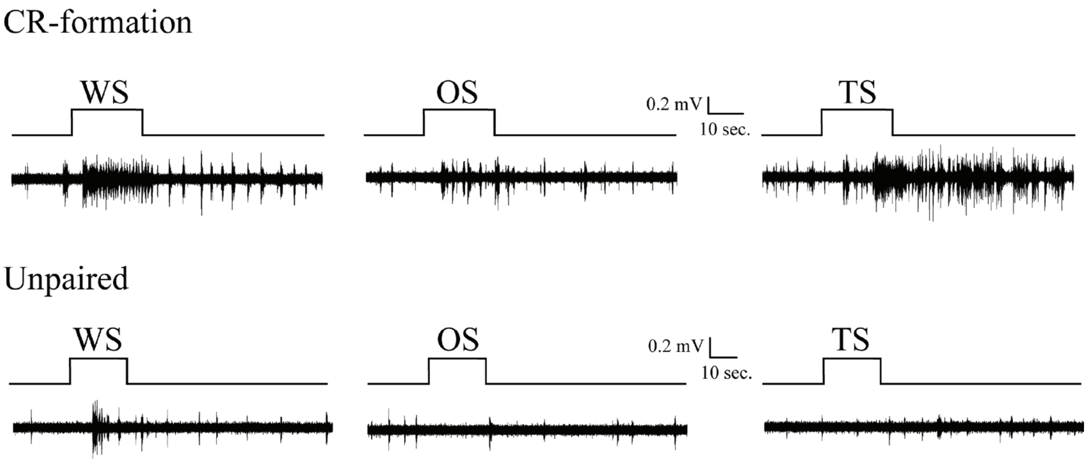

Supplementary Figure 1: Pairing WS, OS and TS leads to the responses of barrel cortical neurons to these signals with the different patterns. A diagram illustrates a recording of local field potentials (LFP) in the mouse barrel cortex. LFPs show that barrel cortical neurons in CR-formation mouse respond to WS, OS and TS. LFPs show that barrel cortical neurons in unpaired control mouse respond to WS. The calibration bars are 0.2 mV and 10 seconds.

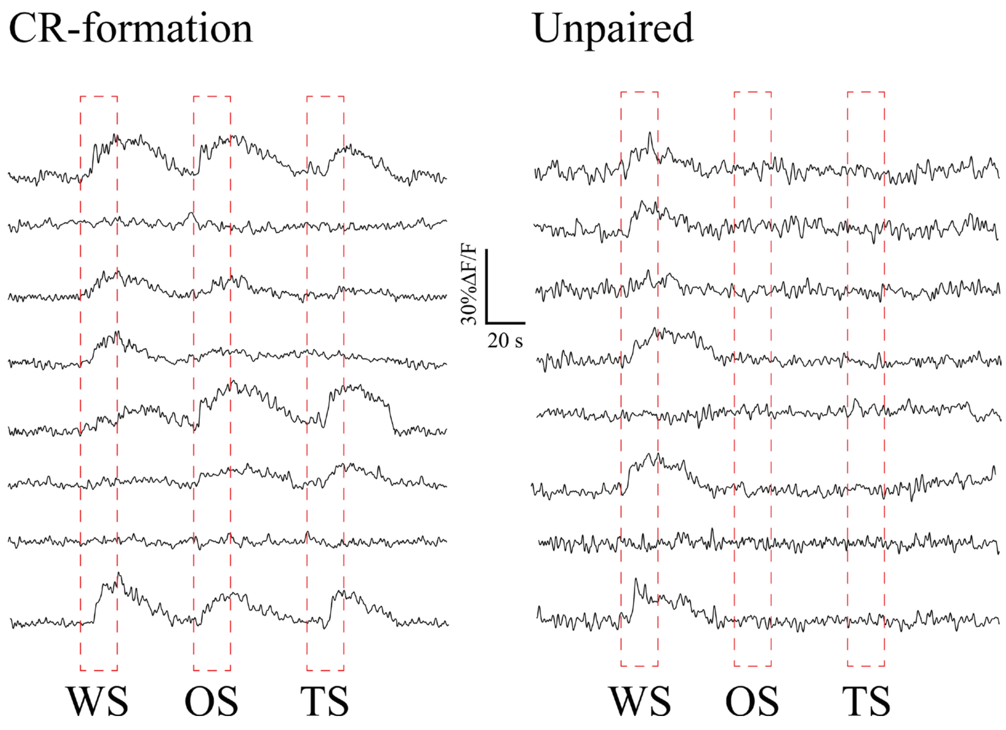

Supplementary Figure 2: Glutamatergic and GABAergic neurons in the barrel cortex respond to the odor signal (OS), tail signal (TS) and whisker signal (WS) after their pairing. Cellular activities were detected by imaging Ca2+ signals under a two-photon microscope in unpaired and CR-formation mice. The digitized Ca2+ signals recorded from barrel cortical neurons in response to WS versus OS and TS from CR-formation mouse (left panel) and CR-formation mouse unpaired control (right). Barrel cortical neurons in CR-formation mouse respond to WS, OS and TS while neurons in unpaired control mouse respond to WS only. The calibration bars are 30% $\Delta F/F$ and 20 seconds.
